# Tocilizumab and IVIG experience during the service follow-up in patients with severe COVID-19 pneumonia

**DOI:** 10.1590/S1678-9946202567028

**Published:** 2025-04-14

**Authors:** Mehmet Ali Tüz, İsmail Türköz, Oytun Aydogan, Emine Gencer, Fadime Özge Aygün-Kaş, Oylum Hunerel, Hande İdil Tüz

**Affiliations:** 1 Balikesir University, Medical Faculty, Infectious Diseases and Clinical Microbiology Department, Balikesir, Turkey; 2 Dörtyol State Hospital, Infectious Diseases and Clinical Microbiology Department, Hatay, Turkey; 3 Uzunmehmet Chest and Occupational Diseases Hospital, Department of Chest Diseases, Zonguldak, Turkey; 4 Private Kapadokya Hospital, Department of Chest Diseases, Nevşehir, Turkey; 5 Zonguldak Atatürk State Hospital, Infectious Diseases and Clinical Microbiology Department, Zonguldak, Turkey; 6 Urla State Hospital, Department of Chest Diseases, İzmir, Turkey; 7 Balıkesir Atatürk City Hospital, Infectious Diseases and Clinical Microbiology Department, Balıkesir, Turkey

**Keywords:** IVIG, Tocilizumab, COVID-19, Severe, Pneumonia

## Abstract

Most SARS-CoV-2 infections are asymptomatic or cause only mild illness, but severe respiratory disease can develop, sometimes requiring oxygen support. Immunopathological damage resulting from an abnormal inflammatory response in patients with severe disease is known to be the main determinant of disease outcome. Studies show that anti-inflammatory therapies work best when used before widespread immunopathological damage has occurred. Similarly, it was thought that intravenous immunoglobulin (IVIG)—holding multiple immunomodulatory effects—would provide clinically favorable results, but recent studies suggest otherwise. Still, the literature shows few studies evaluating the efficacy of IVIG according to the time of administration and there are no studies comparing it with established treatments, such as tocilizumab. In this study, we aimed to evaluate the effects of early administration of tocilizumab and IVIG on clinical outcome in patients with severe COVID-19. Patients with progressive clinical and laboratory deterioration who received tocilizumab or IVIG between 07/2020 and 10/2020 in a public hospital ward were retrospectively evaluated. A total of 74 patients were identified, of whom 29 (39%) received IVIG only and 26 (35%) received tocilizumab only. As a result, patients with severe COVID-19 who received IVIG in early stages of the disease did not have better clinical outcomes regarding mortality, length of hospital stay and ICU admission compared to those who received tocilizumab. Moreover, there is no data to support the use of IVIG in COVID-19 patients with severe disease, as it is associated with more severe side effects and is more expensive than tocilizumab.

## INTRODUCTION

Most SARS-CoV-2 infections are asymptomatic or cause only mild illness, but some patients may develop respiratory issues requiring hospitalization and progress to critical illness with hypoxic respiratory failure requiring ventilatory support^1^. Antiviral drugs are known to be effective in the early stages of infection, within the first 5–7 days after the onset of symptoms, when the viral load is high and the host's immune system is not responding adequately^2^. After this early period, some patients with risk factors progress to severe or critical disease^2^. In patients with particularly critical illness, an excessive and abnormal inflammatory response is thought to be the primary cause of immunopathological damage and is known to be the main factor determining the course of the disease^1,2^. Many studies have shown that anti-inflammatory treatments such as corticosteroids, interleukin-6 (IL-6) inhibitors or Janus-associated kinases (JAK) inhibitors are effective during this period^3,4^. It is recommended that the IL-6 inhibitor tocilizumab and/or the JAK inhibitor baricitinib be used in addition to glucocorticoid therapy in patients with critical illness (patients requiring high flow oxygen or higher oxygen support) and high inflammatory markers^2,5^. In patients with severe but not critical illness (requiring low-flow oxygen support), the administration of the IL-6 inhibitor-tocilizumab and/or the JAK inhibitor-baricitinib is recommended if there is a progressive increase in oxygen support and inflammatory markers during follow-up2. Some studies show that administering tocilizumab in the early stages of severe disease, before immunopathological damage is widespread, leads to better clinical response^2,3,6,7^. These trials suggest that the timing of anti-inflammatory treatments, other than glucocorticoids, may be the reason for the inconsistent results in clinical response to treatment in different trials^6,8^.

IVIG has been successfully used as treatment for a wide variety of inflammatory and autoimmune diseases due to its multiple immunomodulatory effects, such as neutralization of autoantibodies, modulation of synthesis and release of cytokines/chemokines, complement T entry options, regulation of therapeutic compartments of dendritic cells, etc. It was thought that all these immunomodulatory possibilities could be potentially useful to treat COVID-19 patients^9^. In the early days of the pandemic, Chinese studies yielded promising results^10^. A meta-analysis published in January 2022 showed that it did not have a positive effect on mortality in patients with moderate COVID-19, but it did significantly shorten the length of hospital stay^11^. Later, trials and meta-analyses, mostly regarding critical illness (patients requiring high-flow oxygen or higher oxygen supplementation), showed no significant clinical benefit^9^. However, there are a limited number of studies investigating the effectiveness of IVIG in patients with severe but not critical disease (requiring low oxygen support). Moreover, there are no studies in the literature comparing the effectiveness of IVIG with anti-inflammatory treatments shown to be effective for COVID-19, such as tocilizumab.

This study was designed to evaluate the effects of early treatment with tocilizumab and IVIG on the clinical course of patients with severe COVID-19 pneumonia (those who have severe disease but are not critically ill and require low-flow oxygen support).

## MATERIALS AND METHODS

### Study design and population

This study was designed retrospectively and included COVID-19 patients with severe but not critical disease who required low-flow oxygen support and received tocilizumab and/or IVIG treatments^2,12^. The researchers screened the hospital records of patients who were admitted to Zonguldak Atatürk State Hospital, Turkey, from July 5 to October 5, 2020 with a COVID-19 diagnosis and were followed up. Furthermore, the data of the patients who met the inclusion criteria were recorded on the case report form and transferred to the computer environment. The study was approved by the Research Ethics Committee of the Medical Faculty at Zonguldak Bulent Ecevit University (approval N° 2021/01) in Zonguldak.

Inclusion criteria^2,12^;

- Above 18 years of age;
- PCR-positive nasopharyngeal swab or history of close high-risk contact with a PCR-positive individual and whose tomography is compatible with COVID-19 pneumonia;
- Clinical finding: patients receiving oxygen support to maintain SpO2 > 94% after at least 72 h since the start of standard treatment, and who require increased oxygen support as per the application;
- Radiological finding: diffuse ground-glass opacities or infiltrates in more than one lobe compatible with COVID-19 pneumonia recorded on a computed tomography;
- Laboratory: progressive worsening of the following laboratory parameters, measured at 48 h intervals prior to receiving IVIG or tocilizumab, with at least two of the following values: CRP>75 mg/L, ferritin>500 ng/dl, d-dimer>1000 ng/ml, lymphocytes<800/ml;
- Patients who met the criteria and were considered to have developed a cytokine storm and received IVIG and/or tocilizumab treatment during ward follow-up were included in the study.

Patients who required high flow oxygen and ventilatory support to meet spO2>94 prior to starting IVIG or tocilizumab were not included in the study.

### Clinical follow-up and treatment

Patients’ age, sex, total hospital days, ICU days (for ICU patients), comorbidities, treatments received for COVID-19, body temperature, oxygen demand, white blood cell count, lymphocyte, CRP, d-dimer, ferritin, and fibrinogen levels upon admission, on day 3, on day 5, before tocilizumab or IVIG treatment and 48 h after completion of treatment were recorded on the case report form.

Favipiravir was administered from the time of initial diagnosis, and additional low-molecular-weight heparin and supportive care was started during hospitalization. IVIG or tocilizumab treatment was given to patients whose clinical, radiological and laboratory findings described above progressively worsened at least 48 h after hospital admission. IVIG or tocilizumab were randomly selected according to drug availability. Glucocorticoid therapy was subsequently included in the guidelines during the period evaluated in our study. After the guideline change, glucocorticoid therapy was started in patients requiring oxygen. In other words, after glucocorticoid therapy was included in the Turkish COVID-19 guidelines (as of October 9, 2020), dexamethasone 6 mg/day IV was given to patients requiring oxygen support who were enrolled in the study. Tocilizumab was administered in two 400 mg doses 24 h apart and IVIG was administered at a 400 mg/kg/day dose for two days.

### Statistical analysis

Statistical analyses of the study were performed using the SPSS 19.0 package. Descriptive statistics of categorical variables were presented with frequencies and percentages; continuous variables were presented with median, minimum and maximum values or mean and standard deviation. Shapiro-Wilk's and Kolmogorov-Smirnov's tests were used to determine whether continuous variables were normally distributed. Independent t-tests or Mann-Whitney's U-tests were used for group comparisons between two groups of continuous variables. Pearson's Chi-squared test was used for between-group comparisons of categorical variables. In all statistical analyses in the study, results with a p-value < 0.05 were considered statistically significant.

## RESULTS

During the follow-up period, 74 patients diagnosed with COVID-19, under IVIG and/or tocilizumab treatment were identified. A total of 29 (39%) patients received IVIG alone, 26 (35%) received tocilizumab alone and 19 (26%) received both tocilizumab and IVIG.

Of the patients included in the study, 56 (75.7%) were male, their mean age was 63.08±13.537 years, and 63 (85.1%) had at least one comorbid disease. Sixty-seven patients (90.5%) tested positive for COVID via PCR nasopharyngeal swabs, and 7 patients had radiological findings with a history of close high-risk contact. The mean hospital stay was 13.95±7.09 days and the mean ICU stay was 11.65±8.008 days for the20 patients (27%) who required ICU follow-up. All patients were treated with favipiravir. Fever persisted for more than 72 h in 53 patients (71.6%). Sixty-seven patients (90.5%) were discharged, seven patients (9.5%) withdrew. The demographic characteristics and clinical findings of the patients are shown in Table 1 and laboratory data are shown in Table 2.

**Table 1:** General characteristics of all patients.

Age (mean)	63.08±13.537
Gender (Male)	n=56 (%75.7)
Comorbidities	
HT	n=46 (%62.2)
DM	n=23 (%31.1)
CVD	n=29 (%39.2)
COPD	n=7 (%9.5)
CRF	n=8 (%10.8)
Malignancy	n=5 (%6.8)
Hospital admission day	13.95±7.09
ICU admission	n=20 (%27.02)
ICU admission day	11.65±8.008
Oxygenation*	
IMV	n=8 (%10.8)
NIMV	n=2 (%2.7)
HFNO	n=7 (%9.5)
Medical treatment	
Favipiravir	n=74 (%100)
Glucocorticoid	n=57 (%77)
Tocilizumab	n=45 (%60.8)
IVIG	n=48 (%64.9)
Mortality	n=7 (%9.5)
Discharge	n=67 (%90.5)

* Maximum oxygen support requirement; HT = Hypertension; DM = Diabetes mellitus; CVD = Cardiovascular disease; COPD = Chronic obstructive pulmonary disease; CRF = Chronic renal failure; IMV = Invasive mechanical ventilation; NIMV: Non-invasive mechanical ventilation; HFNO = High flow nasal oxygen.

**Table 2:** Laboratory values of all patients at follow-up and pre/post-treatment in patients under immunomodulatory therapy.

		Lymphocytes (/mL)	CRP (mg/L)	Ferritin (ng/dL)	D-dimer (ng/mL)
**Day 1**	Median (min-max)	1060 (340–3110)	70.5 (1–327)	455.5 (16–2000)	343.5 (98–8473)
**Day 3**	Median (min-max)	865 (240–3240)	61 (7–196)	578.5 (26–2000)	392 (82–8863)
**Day 5**	Median (min-max)	815 (180–2280)	30.5 (4–203)	660 (41–2000)	543.5 (103–8753)
**Toci1**	Median (min-max)	800 (240–2140)	81 (15–327)	711 (41–2000)	434 (129–8473)
**Toci2**	Median (min-max)	870 (230–2100)	14 (2–160)	639 (45–1857)	741 (168–8511)
**IVIG1**	Median (min-max)	875 (240–3240)	58.5 (8–258)	712.5 (70–2000)	473 (103–8657)
**IVIG2**	Median (min-max)	825 (180–10300)	22 (2–127)	717 (67–2000)	691 (191–8215)

Toci1 = median and min-max values of prognostic tests immediately before tocilizumab initiation, Toci2 = median and min-max values of prognostic tests 48 hours after tocilizumab treatment was completed; IVIG1 = median and min-max values of prognostic tests immediately before starting IVIG; IVIG2 = median and min-max values of prognostic tests 48 h after completion of IVIG treatment.

When comparing patients who received tocilizumab alone, IVIG alone or both treatments, the groups had a similar age, gender, comorbidities, length of hospital stay and length of ICU stay. The rate of ICU admissions was significantly higher in patients receiving both treatments than in the other two groups (p<0.05) and similar in patients receiving IVIG alone or tocilizumab alone (p>0.05). The group receiving IVIG alone received more glucocorticoids than the group that only received tocilizumab (Table 3). There was no statistically significant difference in mortality in the group receiving IVIG alone compared to the group that only received tocilizumab (p>0.05). Of the 19 patients who received both IVIG and tocilizumab, one in eight patients who received tocilizumab first died, while three in eight patients who received IVIG first died. In these patients, no clinical response was achieved at least 72 h after the end of the first treatment and the start of the other drug. In three patients, the interval between IVIG and tocilizumab treatment was about 24 h.

**Table 3:** Comparison between patients who received tocilizumab and/or IVIG.

	Only Tocilizumab (n=26)	Only IVIG (n=29)	Both Tocilizumab and IVIG (n=19)	p
Age (mean)	64.08±13.371	64.66±13.291	59.32±14.131	>0.05
Gender (Male)	n=17 (%65.4)	n=23 (%79.3)	n=16 (%84.2)	0.293
Comorbidities				
HT	n=16 (%61.5)	n=17 (%58.6)	n=13 (%68.4)	0.788
DM	n=8 (%30.8)	n=11 (%37.9)	n=4 (%21.1)	0.466
CVD	n=8 (%30.8)	n=12 (%41.4)	n=9 (%47.4)	0.505
COPD	n=3 (%11.5)	n=3 (%10.3)	n=1 (%5.3)	0.760
Hospital admission day	13.69±7.745	12.10±4.761	17.11±8.239	>0.05
ICU admission	n=4 (%15.4)	n=6 (%20.7)	n=10 (%52.6)	c
ICU admission day	10.75±8.895	9.67±5.279	13.20±9.343	>0.05
Fever^a^	n=17 (%65.4)	n=22 (%75.9)	n=14 (%73.7)	0.672
Laboratory^b^				
Lymphocytes (/mL)	860 (240–1680)	1000 (240–2550)	610 (320–3240)	>0.05
CRP (mg/L)	76 (20–271)	79 (8–258)	91 (10–327)	>0.05
Ferritin (ng/dL)	745 (41–2000)	748 (70–2000)	711 (95–1937)	>0.05
D-dimer (ng/mL)	463 (129–8473)	474 (83–8657)	397 (103–5598)	>0.05
Medical treatment				
Favipiravir	n=26 (%100)	n=29 (%100)	n=19 (%100)	-
Glucocorticoid	n=13 (%500)	n=26 (%89.7)	n=18 (%94.7)	0.001
Mortality	n=1 (%3.8)	n=2 (%6.9)	n=4 (%21.1)	0.125
Discharge	n=25 (%96.2)	n=27 (%93.1)	n=15 (%78.9)	0.125

a Fever persisting for at least 72 h despite standard care before IVIG/tocilizumab;

b Median and min-max values of prognostic tests immediately before starting IVIG/tocilizumab. Pre-treatment values in the group receiving both;

c P values for intensive care hospitalization rate are given in the text.

**Figure 1:**
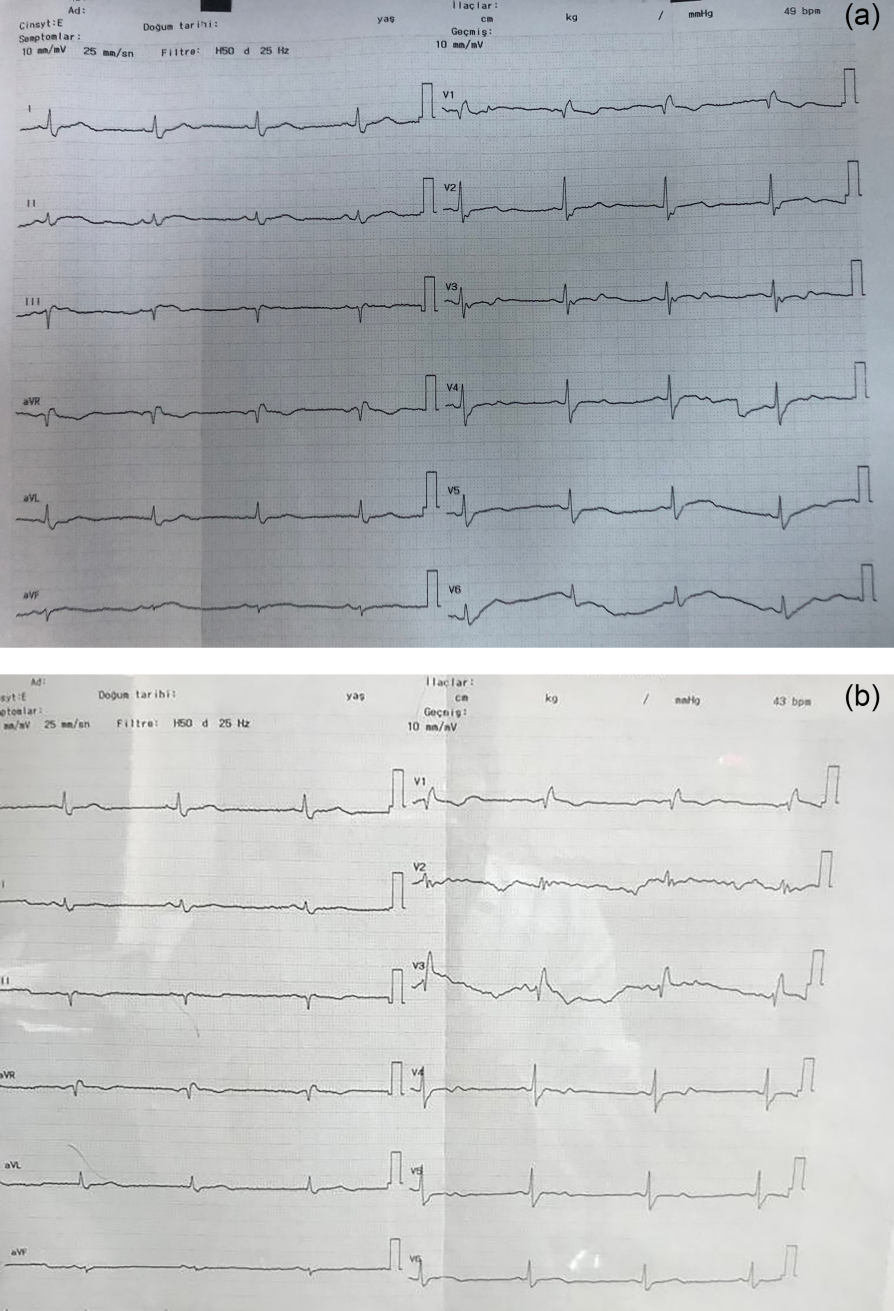
Bradycardia in two patients receiving IVIG.

IVIG treatment was stopped because two patients developed bradycardia during IVIG infusion (Figure 1). The bradycardia persisted for up to 72 h after treatment was stopped. Two patients developed symptoms compatible with transfusion-associated circulatory overload (TACO)-acute pulmonary oedema within the first 6 h after completion of IVIG infusion. No serious drug-related adverse events were observed during or after tocilizumab treatment.

## DISCUSSION

Several studies are investigating the clinical benefits of administering tocilizumab in the hyperinflammatory phase for the treatment of COVID-19, which is now recommended in most guidelines based on evidence that it reduces mortality and limits the need for increased ventilatory support^2,5,13^. However, there are some conflicting results on clinical outcomes between trials^14,15^. It is thought that the main reason for this discordancy is that trials were performed with patient groups that suffered from different disease severities at different times^16^. The REMAP-CAP (Randomized Embedded Multifactorial Adaptive Platform for Community-acquired Pneumonia) study shows reduced mortality in patients receiving tocilizumab within 24 h of admission to the ICU^17^. The EMPACTA (Evaluating Minority Patients with Actemra) study conducted with patients suffering from severe COVID-19 pneumonia that required low-flow oxygen therapy shows that tocilizumab reduces the likelihood of progression to mechanical ventilation or death^18^. Later trials and guidelines generally recommend that immunomodulatory therapies such as tocilizumab should not be administered too early or too late^16^. The most appropriate time of administration is during the first 24 h of follow-up high-flow oxygen therapy in intensive care, or in the event of progressive increase in oxygen requirements and progressive deterioration in prognostic laboratory findings while receiving low-flow oxygen^2,16^.

In the treatment of infectious diseases, IVIG may provide benefits such as limiting hyperinflammation, providing anti-infective properties to treat primary infections, protection against secondary infections and, thus, reduce morbidity and mortality via multiple mechanisms^19^. In theory, early administration of IVIG in the treatment of COVID-19 may be even more important than early administration of other hyperinflammation-targeting therapies to achieve clinical benefits via these multiple mechanisms. Studies conducted before the COVID-19 pandemic show that the response to IVIG use in the treatment of infectious diseases, like sepsis and community-acquired pneumonia, may be related to disease severity and high inflammatory status^19^. Trials investigating IVIG use in the treatment of COVID-19 mostly evaluated critically ill patients receiving high-flow oxygen therapy or higher oxygen support^9,20^. In particular, most trials conducted after the first year of the pandemic show that it does not induce a positive clinical outcome compared with standard care, which may be related to the late administration of the drug and its administration after widespread immunopathological damage^9^. The literature review did not identify any trials that evaluated the use of IVIG in the early stages of the disease, before critical illness, and compared it with other established anti-inflammatory treatments such as tocilizumab. In this study, patients with severe but not critical disease and progressive deterioration of clinical, radiological and prognostic laboratory findings were evaluated. In addition, the clinical outcome of IVIG treatment was compared to tocilizumab treatment. The groups were statistically similar in terms of clinical and laboratory findings. Patients receiving IVIG did not show better clinical outcomes in terms of mortality, length of hospital stay and intensive care unit admission compared to patients receiving tocilizumab.

In COVID-19 trials, serious adverse events in patients receiving tocilizumab or sarilumab did not differ from those in patients receiving usual care^1,2^. Most studies evaluating the side effects of IVIG show that the possibility of side effects per infusion is around 10–15%, with serious side effects occurring in 2% to 6% of patients^21,22^. In our study, no serious adverse events were observed in patients receiving tocilizumab, whereas four patients (8.3%) receiving IVIG experienced serious adverse events.

As mentioned above, IVIG may be potentially beneficial at different stages in the course of COVID-19 treatment via multiple mechanisms, and the timing of its administration may be more important compared to other anti-inflammatory therapies such as tocilizumab^19,20^. In this study, IVIG was administered before the patient reached the critical stage of the disease. Giving IVIG treatment before the severe disease stage may be more effective in limiting disease progression due to the potential antiviral effects of the treatment^19^. However, this may not be a cost-effective approach due to the very large population to which the treatment can be applied. Nevertheless, we believe that further studies focusing on the timing of administration are needed to better assess the benefits of IVIG in the treatment of COVID-19.

Although there is limited data on the possible benefits of combined administration of tocilizumab and IVIG in appropriate patients, studies evaluating COVID-19-related multisystem inflammatory syndrome (MIS-C) in children show that the average hospital stay is significantly reduced, the discharge rate is increased, and complications, like multiple organ failure and respiratory failure, are reduced^23^. In our trial, there were adult patients who were given other drugs when there was no clinical response to one of these two treatments, but these patients did not have better clinical outcomes.

The main limitations of our study were its retrospective nature and the small number of patients. Remdesivir could not be administered due to unavailability. At the beginning of our study, the efficacy of glucocorticoid therapy in the treatment of COVID-19 was not known; the first strong evidence that glucocorticoids reduce mortality and the likelihood of invasive mechanical ventilation was provided by the RECOVERY study in July 2020^24^. During the period evaluated in our study, literature data on glucocorticoid therapy in COVID-19 treatment increased and local COVID-19 guideline recommendations changed, resulting in a difference in the rate of use of these treatments between groups. On the other hand, the REACT study showed that those who received the combination of tocilizumab and glucocorticoids had better clinical outcomes in terms of mortality and invasive mechanical ventilation than those who received tocilizumab alone^15^. In our study, although the rate of concomitant glucocorticoid treatment was higher in the group receiving IVIG, it did not lead to better clinical outcomes compared to the group that received tocilizumab.

## CONCLUSION

In conclusion, severe COVID-19 patients who received IVIG in the early phase of the disease did not show better clinical outcomes in terms of mortality, hospitalization time and intensive care unit admission compared to those who received tocilizumab. In addition, there are no data to support the use of IVIG in COVID-19 treatment due to the higher incidence of serious side effects in IVIG recipients and the significantly higher cost of IVIG treatment compared to tocilizumab.
